# Crosstalk between autophagy and DNA repair systems

**DOI:** 10.3906/biy-2103-51

**Published:** 2021-06-23

**Authors:** Sinem DEMİRBAĞ-SARIKAYA, Hatice ÇAKIR, Devrim GÖZÜAÇIK, Yunus AKKOÇ

**Affiliations:** 1 SUNUM Nanotechnology Research and Application Center, İstanbul Turkey; 2 Koç University School of Medicine, İstanbul Turkey; 3 Koç University Research Center for Translational Medicine (KUTTAM), İstanbul Turkey

**Keywords:** Autophagy, DNA damage, DDR, DNA repair

## Abstract

Autophagy and DNA repair are two essential biological mechanisms that maintain cellular homeostasis. Impairment of these mechanisms was associated with several pathologies such as premature aging, neurodegenerative diseases, and cancer. Intrinsic or extrinsic stress stimuli (e.g., reactive oxygen species or ionizing radiation) cause DNA damage. As a biological stress response, autophagy is activated following insults that threaten DNA integrity. Hence, in collaboration with DNA damage repair and response mechanisms, autophagy contributes to the maintenance of genomic stability and integrity. Yet, connections and interactions between these two systems are not fully understood. In this review article, current status of the associations and crosstalk between autophagy and DNA repair systems is documented and discussed.

## 1. Introduction

Maintenance of cellular homeostasis in living organisms requires a balance between anabolic and catabolic reactions. Various endogenous and exogenous insults lead to the activation of cellular and organismal stress response mechanisms. Macroautophagy (autophagy herein) is one of the major and evolutionarily conserved stress response pathways.

As a catabolic system, autophagy controls degradation of several cellular components, including long-lived proteins, aggregated proteins and even whole organelles (Kocaturk et al., 2019). Hence, autophagy generally contributes to stress resistance and survival of cells. Under certain conditions, excessive autophagic activity was shown to trigger cell death (Oral et al., 2016). Abnormalities in the autophagic activity were associated with various diseases, including neurodegenerative diseases and cancer (Gozuacik et al., 2017; Peker and Gozuacik, 2020) underlining the importance of autophagy for cellular and organismal health, opening the way for autophagy-based treatment approaches (Gozuacik et al., 2014; Bayraktar et al., 2016; Unal et al., 2020). As the key molecule of inheritance, DNA is the essence of life. Exposed to damaging agents and insults, DNA gradually accumulates lesions. All sorts of damages to DNA might potentially result in detrimental outcomes for cells. These lesions also cause loss of genetic information and even trigger genomic instability and rearrangements. Fortunately, in healthy individuals, most of these lesions are repaired by the activation of DNA damage response (DDR) and following DNA damage repair mechanisms. Although autophagic machinery works in the cytoplasm, recent studies pointed out the presence of direct and indirect connections and crosstalk between these stress response systems that are spatially separated.

In this review article, we briefly describe autophagy and DNA repair pathways and dissect molecular and cellular outcomes of interactions and crosstalk between these pathways.

## 2. Mechanisms of mammalian autophagy

Autophagy is a major catabolic process that is observed in all eukaryotic cells. Autophagosomes (or autophagic vesicles) are cytoplasmic double-membrane vesicles that engulf and sequester various cargo molecules, including organelles, proteins and other cellular constituents. Following fusion of autophagosomes with lysosomes, cargo molecules are degraded, and cellular building blocks, such as amino acids, fatty acids and sugars are recycled. As such, autophagy serves as a primary response mechanism that facilitates adaptation to metabolic and other types of stress. Autophagy can be activated by lack of nutrients, growth factor deprivation or endoplasmic reticulum (ER) stress etc., but genotoxic insults such as irradiation, drugs and toxins also trigger autophagy (Eberhart et al., 2016).

Various signaling pathways have been implicated in the regulation of autophagy. Kinase complexes, receptor-mediated events, GTPases, and ubiquitylation-like protein conjugation systems operate in different stages of autophagy. mTORC1 (mammalian target of rapamycin 1) and mTORC2 (mammalian target of rapamycin 2) are the major kinase complexes playing a role in the activation of autophagy. mTORC1 complex is composed of mTOR kinase, mLST8, DEPTOR, Tti/Tel2, RAPTOR, and PRAS40 proteins, whereas mTORC2 contains RICTOR and mSIN1 instead of RAPTOR and PRAS40 proteins (Tian et al., 2019). 

Under basal conditions, mTORC1 orchestrates protein synthesis and growth of cells. In this context, mTORC1 remains active leading to the phosphorylation of autophagy initiation complex proteins ATG13 and ULK1 and blocks autophagy. However, upon nutrient shortage, mTOR complexes are inhibited and autophagy is activated. Autophosphorylation of ULK1 further promotes its activity and induces phosphorylation of several autophagy proteins, including ATG13 and FIP200 (Hosokawa et al., 2009); mTOR complexes are also found to be associated with lysosomes where autophagic cargos are degraded.

Amino acid availability leads to the recruitment of mTORC1 to lysosomes through a mechanism involving amino acid sensing by the RAG family of GTPases. Lysosomal mTORC1 leads to the phosphorylation of the TFE/MITF family of transcription factors and results in their cytosolic sequestration. The abundance of amino acids results in the release of mTORC1 from the lysosomes, thereby its inactivation. Phosphorylation free TFE/MITF transcription factors translocate to the nucleus where they control both the transcription of autophagy and lysosome biogenesis genes (Settembre et al., 2013; Ozturk et al., 2019).

In addition to mTOR, another serine/threonine kinase, AMPK, senses intracellular AMP/ATP ratio and accordingly initiates autophagy. When the level of AMP increases in cells, it binds and allosterically activates AMPK. Binding of AMP to AMPK leads to the activation of the kinase by autophosphorylation as well as by upstream kinases CaMKK and LKB1 (Hawley et al., 1996; Woods et al., 2005) AMPK regulates autophagy in several different ways. AMPK may directly activate autophagy through phosphorylation and activation of ULK1 (Kim et al., 2011). 

On the other hand, phosphorylation-dependent activation of tuberous sclerosis 2 (TSC2) complex by AMPK also regulates mTORC1 activity which further modulates autophagy (Tripathi et al., 2013).

The autophagy process requires the formation of autophagosomes which are double membrane vesicles. Autophagic isolation membranes can either be de novo synthesized or they are derived from existing membrane sources, including ER, mitochondria and their contact sites (MAMs), Golgi membranes or plasma membrane (Ravikumar et al., 2010). Autophagosome nucleation requires activation of another protein complex having a type-III PI3-kinase, VPS34. The PI3 lipid kinase complex contains VPS34, Beclin-1, Atg14, Vps15 and AMBRA1 autophagy proteins. The complex leads to the phosphorylation of membrane-associated phosphoinositol lipids (PI) and converts them into phosphoinositol-3-phosphates (PI3Ps). PI3P lipids on biological membranes facilitate recruitment of lipid-binding proteins (such as DFCP1 and WIPI proteins) onto membranes, marking autophagosome nucleation sites (Carlsson and Simonsen, 2015).

Two ubiquitylation-like conjugation systems are involved in the elongation of autophagic membranes: The ATG12-5-16 system and the ATG8/LC3-lipid conjugation system. First, ATG7 acts as an E1-like enzyme and activates ATG12. Then ATG12 conjugates with ATG5 with the help of the E2-like enzyme ATG10. Following the conjugation of ATG12 and ATG5, the complex interacts with another autophagy protein ATG16L. Forming ATG12-5-16 complex performs an E3-like function in the second conjugation system (Kuma et al., 2002; Fujita et al., 2008). The second system leads to the activation of ATG8/LC3 proteins (MAP1LC3 or simply LC3 protein, GATE-16 and GABARAP1/2 proteins) through the involvement of E1-like enzyme ATG7 and E2-like enzyme ATG3. Of note, before lipid conjugation, ATG8/LC3 proteins should be primed by ATG4 proteins through a C-terminal cleavage (Li et al., 2011). Once ATG8 proteins are activated, the ATG12-5-16 complex from the first system serves as an E3-like ligase and facilitates the conjugation of ATG8 proteins to lipid molecules, such as phosphatidylethanolamine (PE). Lipidated ATG8 proteins promote isolation membrane expansion and autophagic vesicle completion (Lystad and Simonsen, 2019). Moreover, recent data indicate formation of mTOR-inhibition-sensitive higher molecular weight regulatory complexes, including ATG12-5-16 and the adaptor protein GNB2L1 (RACK1) as key components (Erbil et al., 2016).

In the case of selective autophagy, cargo-autophagosome interaction requires specific receptor proteins containing LC3-interacting motifs (LIR motifs) and ubiquitin-binding domains (UBA). SQSTM1/p62, NBR1, NDP52 (also known as a CALCOCO2), OPTN, NIX (also known as BNIP3L) were documented as cargo selective autophagy receptor proteins (Johansen and Lamark, 2020)

Autophagic cargos have to be degraded to finalize their journey. Autophagosomes fuse with lysosomes, and the resulting compartments, autolysosomes are responsible for degradation. The fusion process requires several proteins and complexes, such as SNARE proteins (e.g., syntaxin 17 (STX17), SNAP29 and VAMP8, integral lysosomal proteins (e.g., LAMP-2) and RAB proteins (e.g., RAB5 and RAB7) (Bento et al., 2013). In autolysosomes, cargos are degraded to their building blocks, they are recycled and reused by cells, allowing resistance to stressful conditions and survival.

Many of the abovementioned autophagy mechanisms and pathways are also activated during genotoxic stress. Even direct protein-protein interactions have been reported between these two stress responsive systems.

## 3. Mechanisms of DNA damage response (DDR) and DNA repair

Depending on causative factors, the type and impact of damage on DNA may vary. Severity of the DNA damage is responsible for the decision of cellular response. DDR is a complex cellular mechanism which involves the activation of several molecules that are stimulated in response to DNA damages (Matt and Hofmann, 2016). Ataxia-telangiectasia mutated (ATM), ATM and RAD3-related (ATR), and DNA-dependent protein kinase catalytic subunit (DNA-PKcs) are the major regulator of DDR (Menolfi and Zha, 2020). DDR and following DNA repair signaling initiated with the recognition of the damage involves activation and recruitment of various factors according to type of damage.

Damaged DNA becomes a subject for DNA repair pathways. At least five major distinct types of DNA repair mechanisms, base excision repair (BER), nucleotide excision repair (NER), mismatch repair (MMR), nonhomologous end-joining (NHEJ), and homologous recombination (HR) have been established. Different factors were shown to take place to the decision of the type of repair pathways. Although studies were described possible intersections and spatio-temporal activation of those pathways, yet activation of which major repair mechanisms depend more on type of DNA damage. 

Base damages can be either single or multiple and bulky. In general, BER responsible for the removal of an abasic single base damage, however multiple and bulky base damage repairs by NER. MMR corrects multiple and bulky base mismatches and also replication errors. In addition to base damages, damaging agents may also lead breaks on DNA strands. Single or double strand breaks are repaired either by Single strand break repair pathways (SSBRs) or double strand break repair pathways (DSBRs) (Jackson and Bartek, 2009).

### 3.1. Damaged base-assisted repair mechanisms

**Figure 1:**
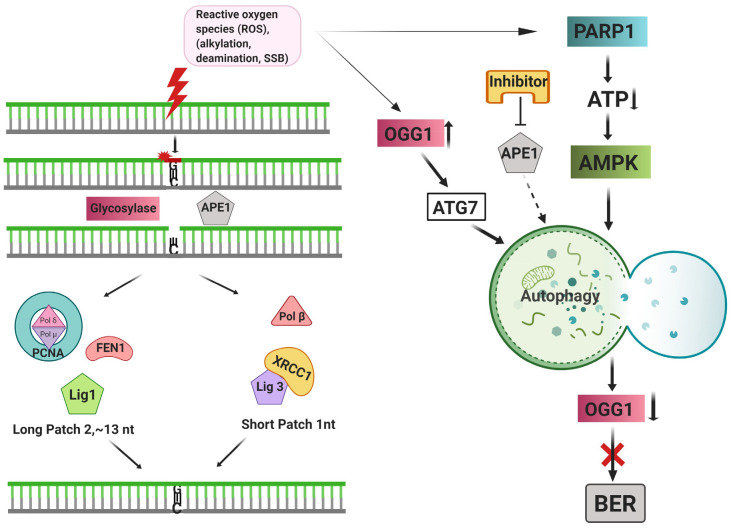
Schematic representation of the BER pathway and its crosstalk with the autophagic process.

Three major base-assisted repair mechanisms have been discovered in mammalian cells. Base excision repair (BER) is one of the first main base-assistedrepair systems (Robertson et al., 2009). Nonbulky, single base DNA lesions bearing only small chemical changes like alkylation, oxidation or deamination are specifically repaired by BER. A specific DNA glycosylase enzyme functions in the detection and removal of the damaged base in this conserved mechanism. Following detection, the damaged base is flipped out of the DNA helix (Figure 1). In this way, even small base changes can be detected sensitively. 

Two different glycosylases have been addressed in this system depending on their function. First, monofunctional glycosylases such as UNG (uracil-N glycosylase), SMUG1 (single-strand-specific monofunctional uracil DNA glycosylase), MBD4 (methyl-binding domain glycosylase 4), TDG (thymine DNA glycosylase), MYH (MutY homolog DNA glycosylase) and, MPG (methylpurine glycosylase) only exhibit glycosylase function. The second type of glycosylases such as OGG1 (8-oxoguanine DNA glycosylase), NTH1 (endonuclease ΙΙΙ-like), and NEIL1 (endonuclease VΙΙΙ-like glycosylase) have an intrinsic 3’AP lyase activity in addition to their glycosylase activity. The final step of BER is perpetuated with the same mechanism regardless of the type of glycosylase and its function on DNA lesion. Once AP sites were produced, AP endonuclease 1 (APE1) taking place whose activity resulted in 3′-hydroxyl and a 5′-2-deoxyribose-5′-phosphate (5′-dRP) through cleavage of DNA backbone from 5’. 

Subsequently, this exposed 3’-hydroxyl is attacked by DNA polymerase β (Polβ) and the gap is fulfilled by the guidance of a template-directed synthesis. In addition, AP sites can form as a single nucleotide or 2-13 nucleotides long depending on the length of the processed nucleotides by a polymerase. Therefore, the length of the filled nucleic bases may alter the following process. In single-nucleotide changes, 5′-dRP cleaved by the intrinsic dRP-lyase activity of Polβ in single nucleotide whereas, flap endonuclease 1 (FEN1) takes place for the removal of the displaced 5′-flap structure in long patch repair by BER (Lee and Kang, 2019).

**Figure 2:**
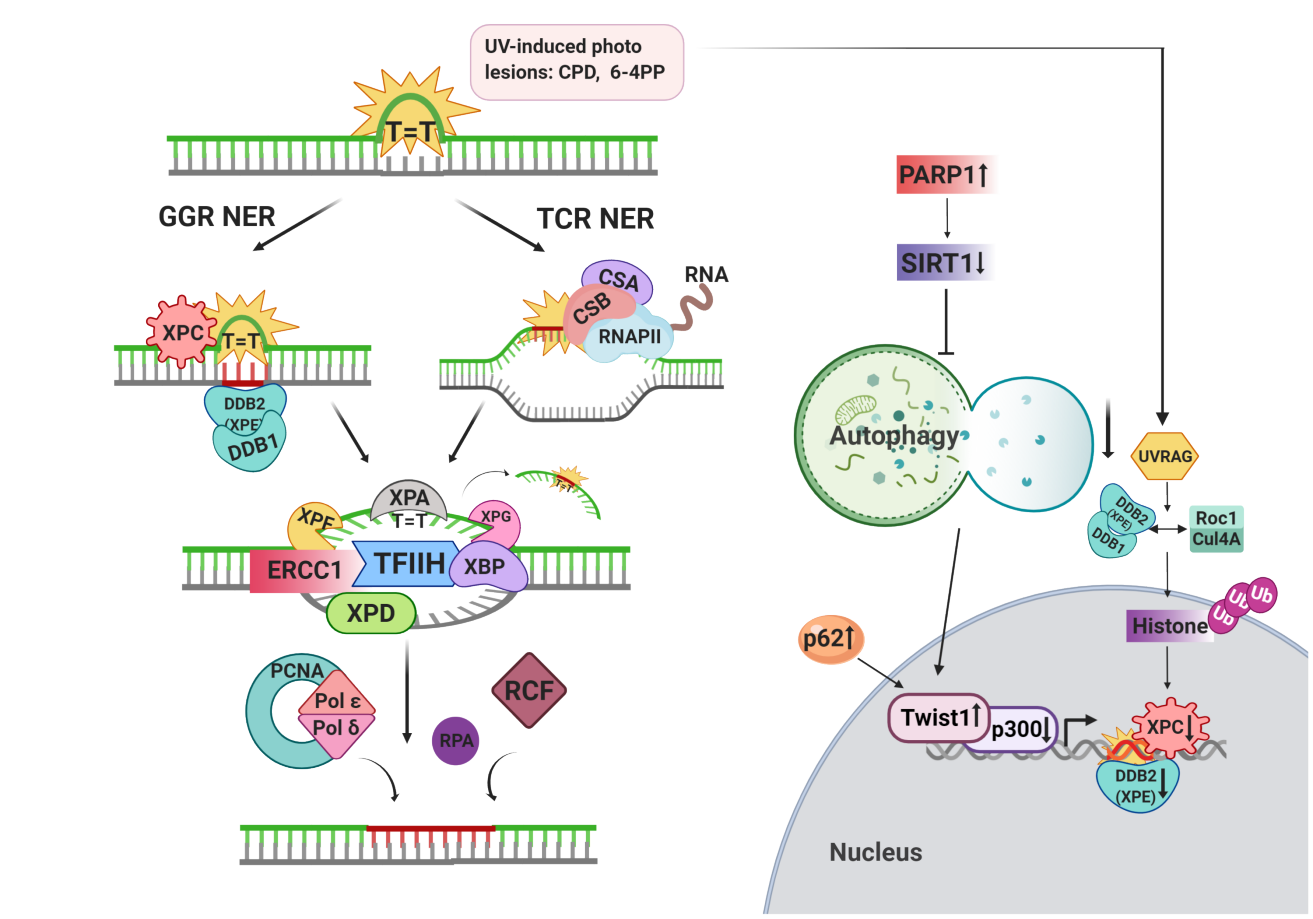
Schematic representation of the NER pathway and its crosstalk with the autophagic process.

Nucleotide excision repair (NER) is one of the second base-assisted repair systems. Under physiological conditions, bulky DNA adducts (e.g., thymidine dimers) on the DNA strand which alter the helix structure and block the proper functioning of polymerases are primarily repaired by NER (Gillet and Schärer, 2006). For instance, UV-induced cyclobutane pyrimidine dimers (CPDs) and 6-4 pyrimidine-pyrimidone photoproducts (6-4PPs) are the major DNA lesions being targeted by the NER system (Menck and Munford, 2014). NER mechanism consists of sequential activation of different protein complexes (Figure 2). At first, DNA damage is detected and the DNA double helix is unwinded. Following detection, each end of the lesions is cut, and the damaged strand is eliminated. Furthermore, the gap between the damaged strand of DNA is filled by polymerases. Then, end ligation of corrected DNA occurs.

NER system can be accomplished by two subpathways: global genome repair (GGR) and transcription-coupled repair (TCR). The location of lesions and protein complexes determines the type of these subpathways to be activated in NER. XPC-RAD23B complex (Xerodermapigmentosum complementation group C-human homolog B of S. cerevisiae RAD23) searches and detects the lesions throughout the genome and promotes GGR (Fagbemi et al., 2011). Upon binding of this complex to the opposite strand of the damaged region, transcription factor II H (TFIIH) is recruited to the site and GGR-mediated repair occurs. However, in the TCR pathway, lesions forming on actively transcribed genes result in the stalling of RNA polymerase II. Lesion sites are mainly detected by the Cockayne syndrome A (CSA) and Cockayne syndrome (CSB) proteins. TFIIH transcription complex recruitment process is shared between two subpathways. Recruitment of TFIIH complex leads to an unwinding of DNA by forming a bubble (~30bp) and subsequent recruitment of XPA (xeroderma pigmentosum complementation group A) and replication protein A (RPA) proteins. Both edges of the damaged strands are cut by endonuclease activity of XPF/ERCC1 (xeroderma pigmentosum complementation group F/Excision repair crosscomplementation group 1) and XPG (xeroderma pigmentosum complementation group G) proteins. Subsequently, DNA polymerases δ and ε fill the gap with the help of replication factor C (RFC) and PCNA (Mocquet et al., 2008). Finally, forming nicks are ligated by LIG1 and LIG3 to finalize the repair of damaged DNA.

DNA mismatch repair system (MMR) is the third and last well-conserved base repair mechanism. MMR mainly and specifically targets base-base mismatches and mispairing of insertions or deletions during replication or recombination (Li, 2008). Thus, MMR is considered as an urgent postreplicative repair mechanism. During the DNA replication period, compromised DNA polymerases proofreading activity is restored by MMR to some extent (Guarné and Charbonnier, 2015). 

**Figure 3:**
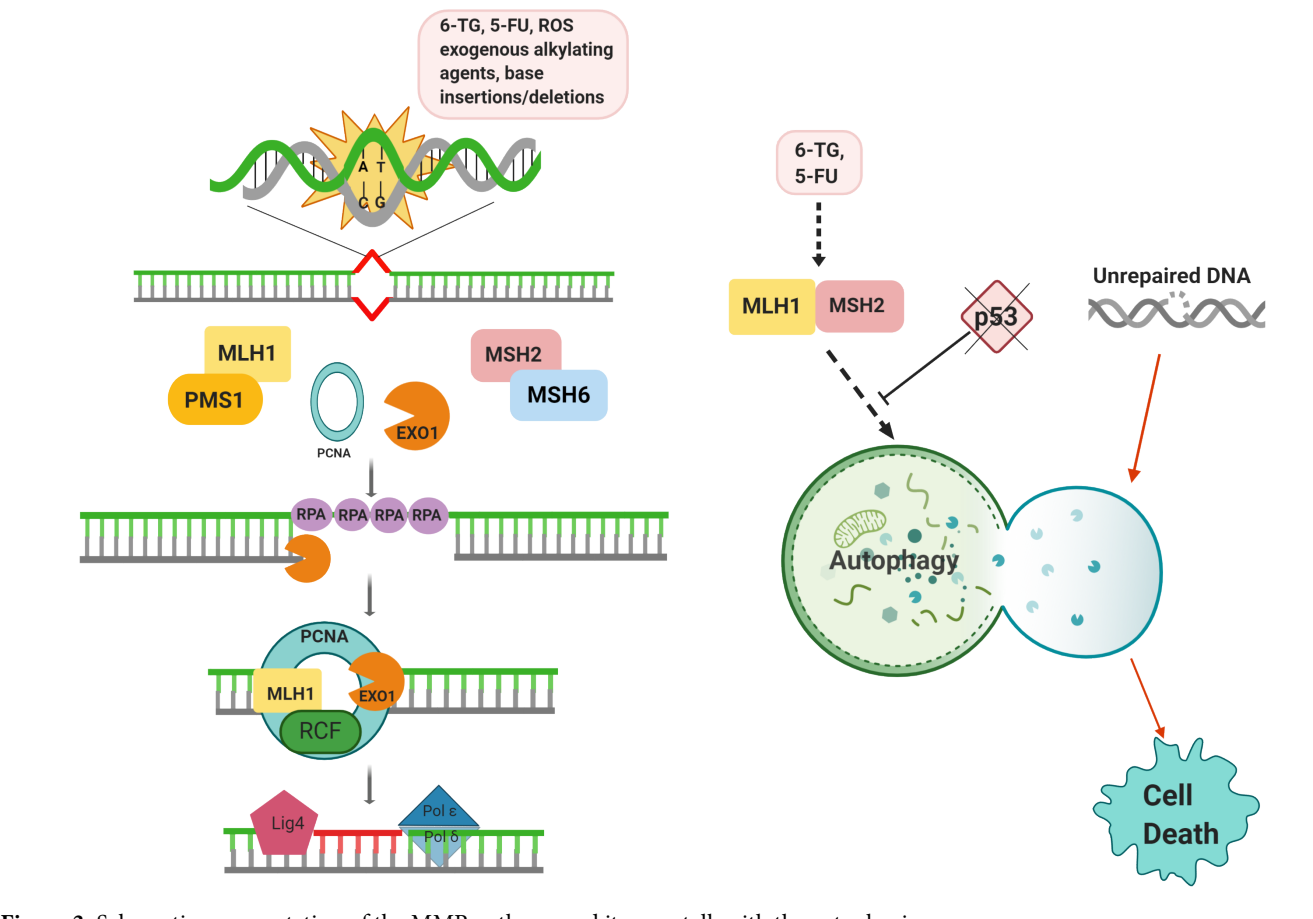
Schematic representation of the MMR pathway and its crosstalk with the autophagic process.

Moreover, rather than replication stress, exposure to endogenous and exogenous DNA damaging substances can also cause base alterations to be repaired by MMR (Martin et al., 2010). Canonical MMR system functions in line with replication machinery and is classified into four key phases (Figure 3). In the first step, mispaired bases (A:G, T:C) are detected. Then, the nascent strand carrying the misincorporated nucleotide is determined. Subsequently, dislocation or endo-/exonucleolytic digestion of the nascent strand occurs. Finally, a mispaired DNA sequence is corrected with ligation and resynthesis. During MMR mediated correction of the errors, parental strand and newly synthesized strand are differentiated by damage detectors of MMR and a misincorporated segment is labeled for removal with a poorly understood mechanism (Guarné and Charbonnier, 2015). By using parental DNA as a template, the base sequence of newly synthesized DNA is corrected (Martin et al., 2010). These errors must be rescued until the end of the S phase, otherwise unrepaired products give rise to microsatellite instabilities or frameshift mutations following cell division cycles (Kinsella et al., 2009; Guarné and Charbonnier, 2015).

In order to sense damage, two complexes; MSH2:MSH6 and MSH2:MSH3 are formed based on the type of damage. Base additions and the small insertion/deletions are detected by MSH2:MSH6 complex. However, insertion/deletion loops up to 10 nucleotides are recognized by MSH2:MSH3 complex (Martin et al., 2010). Moreover, the second MSH complex including MutL homolog 1(MLH1) and its binding adaptors, PMS1 or PMS2 (postmeiotic-segregation increased protein) are recruited to the recognition area. Similar to the sliding clamp concept, the MSH and MLH complexes slide over DNA until they encounter any single-strand DNA gap (Martin et al., 2010). 

In parallel, a replication protein A (RPA) acts as a flagger and recruits another stabilizing protein (RFC) and a progressivity factor (PCNA) to bind and protect the damaged DNA region. Recruiting of all these proteins acts as an attraction point for the arrival of the next complexes. Confirmation and identification of an error in the daughter strand are accomplished, when MutL complex meets the cluster at the single-strand gap. After the definitive identification of the gap by MutL it allows the recruitment of DNA exonuclease (Exo1) into the repair site for the removal of the damaged region. MLH:MSH complexes stay bound until the end of the excision period. A specific polymerase, Pol δ synthesis new DNA in the excised region. Similar to the MLH:MSH complex, PCNA also remains onto DNA at the end of the synthesis of new DNA to provide the sliding activity of the complex over the new sequence and check the progress. In the last step of repair, joining of new DNA to the previous daughter strand was performed by Ligase I (Martin et al., 2010). MMR corrects errors in the daughter strand but errors may also occur in template strands as well. In this case, intrinsic problems occur and cause DDBs.

### 3.2. Strand breaks-assisted repair mechanisms

Two major strand breaks-assisted repair mechanisms have been discovered in mammalian cells. Single strand breaks generally caused by oxidative damage or as an error of DNA topoisomerase enzyme which further cause collapse of DNA replication, stall ongoing transcription and activates PARP1. In long patch SSBR pathway, SSBs detected by PARP1 and caused following poly (ADP) ribosylation on DNA. Tagged damage then processed by apurinic-apyrimidic endonuclease 1 APE1, PNKP (polynuceotide kinase 3′-phosphate) and aprataxin (APTX) (Lee and Kang, 2019). After that FEN1 removes the damaged 5’ and leads to the production of ssDNA gap which filled by POL β, in combination with POL δ/ε. As a last step ligation facilitated by LIG1 with the presence of PCNA and XRCC1 (Lee and Kang, 2019).

In the short patch SSBRs, similar end processing happens like long patch SSBR, yet it is taking place when BER generated SSBs are recognized by APE1. Moreover, gap filling process only requires POL β rather than other polymerases and ligation facilitated by LIG3 (Lee and Kang, 2019).

DNA damages not only differ from each other physically but also sources and mechanisms of them are distinctly different. DSBs naturally occur by well-defined mechanisms such as, V(D)J recombination or meiosis at a particular region of the genome (Schatz and Swanson, 2011). Moreover, some of the intrinsic e.g., stalled or collapsed replication forks or extrinsic e.g., IR and chemotherapeutic agents are shown to cause DSBs experimentally (Schipler and Iliakis, 2013).

**Figure 4:**
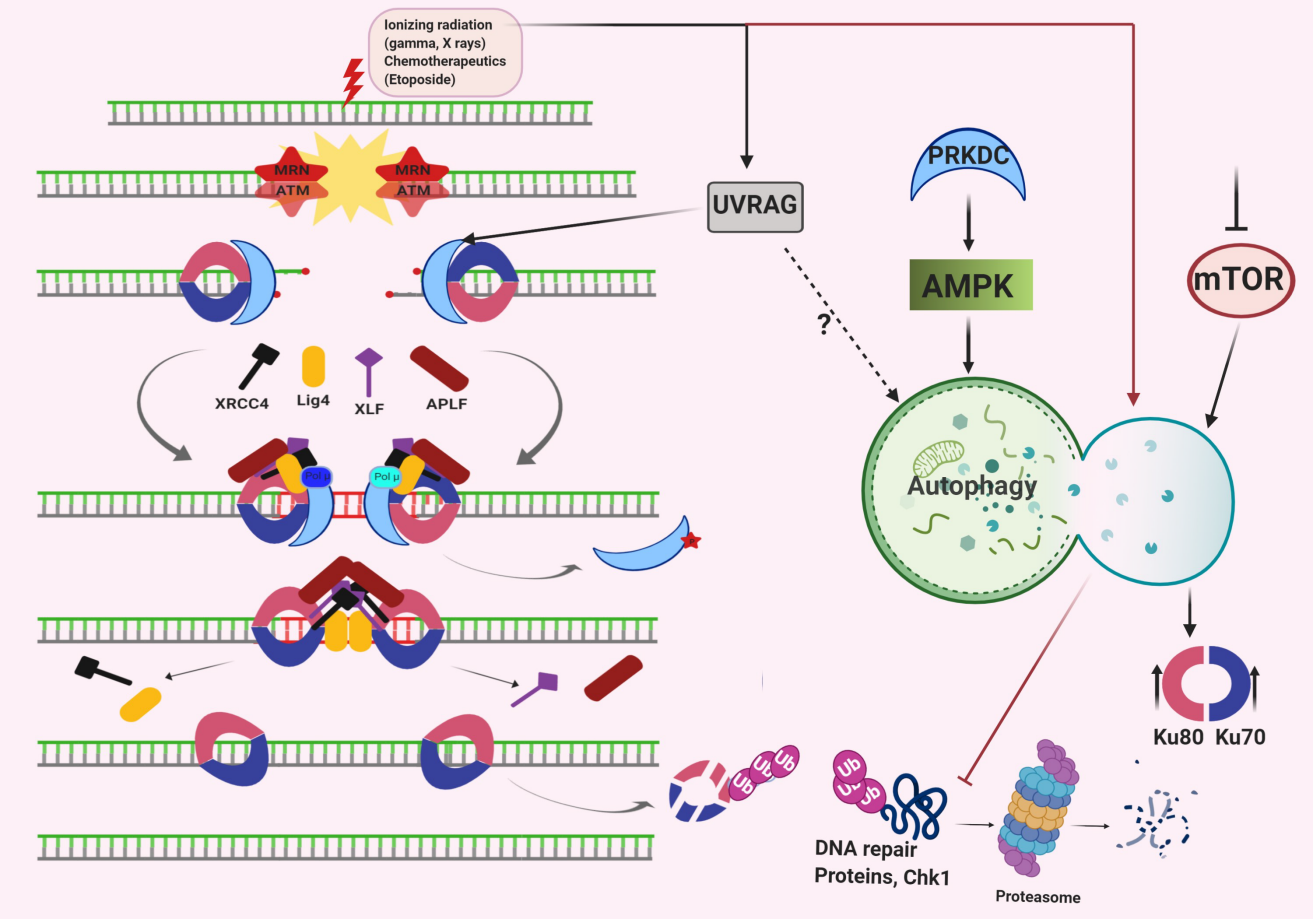
Schematic representation of the HR pathway and its crosstalk with the autophagic process.

Homologous recombination repair (HRR) is one of the well-known and highly conserved DSB repair system. Apart from SSB-associated repair mechanisms, HRR shows a high level of accuracy with the presence of identical DNA copy. HRR initiates with the production of 3’-single-stranded DNA overhangs following the recognition and end processing of double-strand breaks (Figure 4). This step is highly coordinated by multiprotein complexes that support helicase and nuclease activity. Through the activity of multiprotein complexes, strand exchange proteins of HRR; RecA or RAD51 loaded onto the handled single-stranded (ss) DNA (Spies and Kowalczykowski, 2005). This initial step is calledpresynapsis in which monomers of RecA/Rad51 proteins create a helical nucleoprotein fiber by polymerizing onto ssDNA and it is used for homology search. After homology search, non-homologous and homologous links are formed and the next step called synapsis takes place. When there is a homologous pairing between a region of the RecA-ssDNAsegment and dsDNA, strand exchange occurs and a joint molecule, called d-loop, is built. Formation and dissociation of d-loop are tightly controlled by mediator proteins and this molecule acts as a precursor for downstream pathways of HR (Kanaar et al., 2008). Depending on d-loop stability, HR subpathways are determined. For example, the nascent d-loop extension favors one of the HR subpathways, while disassembling of the d-loop causes interruption of the HR reaction. Of note, synthesis-dependent strand annealing (SDSA) promotes disassembly of extended d-loop as an antcrossover mechanism (Tham et al., 2016).

In the double strand break repair (DSBR) subpathway, branch migration that extends the heteroduplex region occurs between the invaded strand and template strand. In this way, it forward catching of second 3’end and formation of a secondary d-loop. During the branch migration, lost information of damaged DNA is brought back with DNA synthesis on the homologous template. Both 3’ends are brought together with DNA ligase which gives rise to recombination byproduct including double Holliday junction. Double Holliday junctions can be resolved by site-specific endonucleases and lead to the formation of crossover (CO) or noncrossover products based on cleavage position. In the SDSA process, instead of capturing the second 3’end, extended initial d-loop is disassembled. Thus, it permits annealing of strands between the two 3’ends of damaged DNA and DNA synthesis leads to the recovery of lost information. In this way, CO event cannot be observed. On the other hand, break-induced repair pathway (BIR) uses a second DNA molecule for an extended region to copy lacking information, but it never uses a second 3’end (Tham et al., 2016).

**Figure 5:**
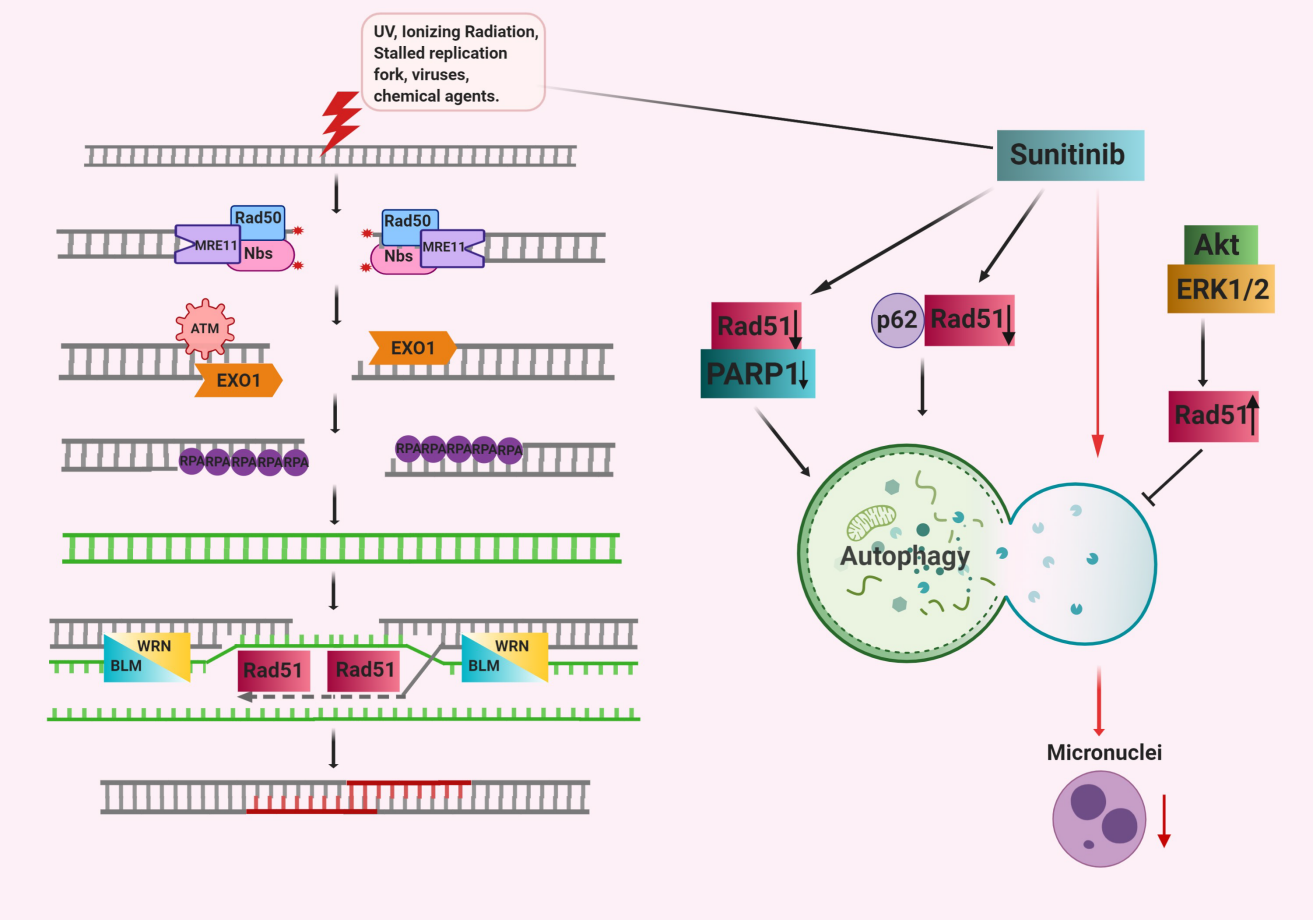
Schematic representation of the NHEJ pathway and its crosstalk with the autophagic process.

In contrast to homologous recombination, NHEJ is an error-prone and imprecise mechanism in which DNA break sites are repaired to provide chromosomal integrity (Takata et al., 1998). NHEJ system is modulated by several proteins including Ku70, Ku80 and a DNA-dependent protein kinase catalytic subunit (DNA-PKcs), XRCC4, DNA ligase IV, Artemis and XLF (Lieber et al., 2010). NHEJ activity is initiated by the binding of Ku70/80 heterodimer on DNA damage site to flag the damaged region (Figure 5) (Waters et al., 2014). Following damage recognition, DNA-PKcs binds to the Ku proteins and this complex further recruit nucleases, polymerases and ligases to the damaged site (Lieber, 2008). In the presence of DNA ends, Ku proteins undergo a conformational change and only in this way they can make a stable complex with DNA-PKcs (Yaneva et al., 1997). 

Under this condition, the established complex drives the interaction of Ku proteins with DNA polymerases µ and λ, and the XRCC4-DNA ligase IV complex (Chen et al., 2000). With the help of these interactions, the ends of DNA are brought together. Subsequently, DNA-PKcs represent its kinase activity to phosphorylate various repair proteins and auto-phosphorylate itself (Gottlieb and Jackson, 1993; Chen et al., 2000). Of note, most of the time these complexes show high flexibility and may allow associating with other damaged regions for activation of NHEJ pathway on those sites as well (Bétermier et al., 2014)

In the above section we mentioned the mechanisms of both autophagy and DNA repair systems in detail. Characterization and contribution of autophagy mechanism upon DNA damage is crucial. There are several articles emphasizing the role of autophagy in genome maintenance. In the following section we will discuss the involvement of autophagy in genome maintenance by providing examples from literature.

## 4. Autophagy and genome maintenance

As a cellular degradation process, autophagy leads to the elimination of damaged organelles and proteins, including mitochondria and cancer-relevant proteins, hence, it limits proteotoxicity and oxidative burden. As such, autophagy functions as a mechanism that contributes to protection from DNA damage. In line with this, most agents causing DNA damage were shown to activate autophagy. ROS are highly active molecules and generated as byproducts of metabolic processes that are generally associated with mitochondria and peroxisomes. Although ROS contributes to cellular signaling pathways in the cell, excess ROS levels and reduced detoxification threaten proteins, lipids and genetic material in cells. ROS can result in direct effect on DNA which causes the formation of 7,8-dihydro-8-oxo-guanine (8-oxo-G). Accumulated nonrepaired 8-oxo-G has increased the chance of mispairing with adenine leading to genomic instability (Van Loon et al., 2010). Moreover, ROS may also target the phosphodiester bond to create DSB and trigger chromosome alterations or cell death (Kinner et al., 2008)

Through mechanisms summarized above, autophagy is necessary for the limitation of ROS generation and further genomic instability. For instance, accumulated centrosome abnormalities, increased chromosome numbers were detected in autophagy-deficient cells (Mathew et al., 2007). Loss of autophagy genes including *atg5* and *atg7* in different mice models resulted in the accumulation of damaged mitochondria and ROS which further led to DNA damage and cell death (Mortensen et al., 2011; Komatsu et al., 2011). Autophagy compromised cells are no longer able to stabilize the levels of ROS, accumulated p62 and eliminate the number of damaged mitochondria through mitophagy to alleviate increased DNA damage. Autophagy deficiency caused by Beclin-1 heterozygosity resulted in genomic instability following ROS accumulation and DNA damage to drive breast cancer tumorigenesis (Karantza-Wadsworth et al., 2007). Controversially, aberrant DNA damage and elevated autophagic activity were documented to cooperatively regulate the progression of the malignant form of pancreatic cancer (Yang et al., 2011). Interestingly, in some cases, loss of autophagic activity causes DNA damage and genomic instability. Then, loss of tumor suppressor genes such as p53, results in cell cycle arrest and checkpoint inhibition to hijack the presence of DNA damage and to continue cell division (Yang et al., 2014). 

As such, autophagy and DNA damage in cancer may not always act on the same pathway yet they are not mutually exclusive.

In line with this, autophagy associated proteins were also found to modulate DNA damage response. p53 induces autophagy through the upregulation of damage-regulated modulator of autophagy-1 (DRAM1) (Crighton et al., 2006). In another example, p53 was shown to modulate autophagy through death-associated protein kinase-1 (DAPK1) (Martoriati et al., 2005). DAPK1 is one of the major kinases found to be associated with two distinct cell death mechanisms orchestrating both caspase activation and autophagy in response to ER stress (Gozuacik et al., 2008). 

p18-CycE, a cyclin E fragment, which is identified in hematopoietic cells underwent DNA damage-induced apoptosis. Chronic expression of the fragment caused aberrant autophagy and its turnover regulated by autophagic activity. Furthermore, p18-CycE reported to interact with Ku70, NHEJ components, and stabilized the protein in cytosol upon DNA damage and induced cellular senescence in the lung cancer cell (Singh et al., 2012). SQSTM1/p62, which is an autophagy receptor protein, accumulated in cells and translocated to the nucleus upon autophagic deficiency. Nuclear p62 was found to bind E3 ligase RNF168s and blocked its function on DNA repair. DNA damage-mediated ubiquitination of H2A regulated by RNF168s and facilitated the recruitment of DDR and repair factors on DSBs sites (Wang et al., 2016). A serine/threonine kinase Lkb1 was phosphorylated by ATM and facilitates activation of AMPK which turns on the inhibition of mTORC1 through TSC2 complex (Alexander and Walker, 2011). 

PARP1 is one of the main enzymes which was recruited on DNA lesion and whose activation led to the consumption of NAD+. DNA damage-induced activation of PARP1 has manifested an energy crisis which has been sensed by AMPK later. In addition, this response was associated with cellular ROS accumulation and the cytoplasmic pool of ATM. Loss of PARP1 restrained mTOR activity and delayed autophagy (Rodríguez-Vargas et al., 2012). In another study, IR-induced prolonged DSBs and genomic instability phenotype have also been associated with loss of autophagy (Ito et al., 2005).

The role of autophagy in chemotherapeutics-induced DDR and following cell death is another important subject in this context. Majority of DNA damage-inducing drugs e.g., etoposide, have been shown to induce autophagy. For instance, genetically engineered MEF cells lack fundamental proapoptotic genes *Bax* and *Bak* exhibit elevated autophagy and autophagy-dependent cell death following etoposide and staurosporine exposure (Shimizu et al., 2004). Other DNA damaging agents, topotecan and cisplatin have been found to activate ATM. Activation of ATM leads to the phosphorylation of the PTEN protein at Ser113 residue and facilitates the translocation of PTEN to the nucleus. PTEN nuclear localization led to the phosphorylation of JUN, followed by increased SESN2 (Sestrin2) gene expression. AMPK is activated by SESN2 and induced autophagy in both cervical and lung cancer cells (Chen et al., 2015).

In recent years, nanoparticles have been utilized as a targeted therapy against cancer cells. Nanoparticle loaded DNA damaging drugs including doxorubicin and cisplatin have been used to target and eliminate cancer cells specifically (Gozuacik et al., 2014). Doxorubicin loaded NPs shown to target cancer cells and induced DNA damage by the controlled release of the drug (Yar et al., 2018). Besides receptor-specific targeting of lung cancer cells exhibits an elevated level of autophagy upon damage. Moreover, autophagy deficiency further promotes apoptosis following 5-FU-loaded NPs (Duman et al., 2019). Cisplatin and 5-FU induce both autophagy and DNA damage. Absence of autophagy provides favorable conditions through prolonged DNA damage and increases the potency of chemotherapeutic agents (Claerhout et al., 2010; Duman et al., 2019). In other instances, heavy metal exposure has been found to induce DDR upon DNA damage. Upon Cadmium (Cd), which is a heavy metal, exposure ROS level shown to be elevated, followed by DNA damage and activation of autophagy has been documented in mouse spermatocyte-derived cells. Cd-mediated DNA damage-induced autophagy through the inhibition of mTOR by AMPK which activated upon the increased level of ATM (Li et al., 2017).

As discussed above, as a cellular degradation process, autophagy plays a role in the elimination of genotoxic stresses including ROS and damaged mitochondria. Unfixed damage of DNA results in genomic instability which is further associated with cellular senescence or cell death. Besides, autophagy may provide the energy required for supporting cell cycle arrest and maintaining DNA repair during DDR. Moreover, several repair proteins have been found as a target of autophagy. Therefore, restoration of DNA damage through modulating autophagy may serve as a target to improve those cellular catastrophizes. Understanding the role of autophagic activity or autophagy-facilitated modulation of DNA repair effectors have not been studied elaborately (as summarized in Table). In the following section, we will discuss in detail the main DNA repair mechanisms and their crosstalk with autophagy.

**Table:** The list of studies conducted on autophagy and DNA repair systems.

Cell line/tissue and organism	Drug/genetic modification	Repair mechanism	DNA repair-associated target	Autophagy status	Quantification of DNA damage	Reference
Ampk -/- and WT MEFs	UVB	NER	XPC	N.D.	Slot blot assay of CPD and 6-4PP	(Wu et al., 2013)
Atg5 -/- ; Atg7 -/- and WT MEFs, HaCaT	UVB	NER	XPC	Inhibited	Slot blot assay of CPD and 6-4PP	(Qiang et al., 2016)
Hs294T, A2058	Cisplatin	NER	XPA	Activated	PARP1 activity	(Ge et al., 2016)
Primary human fibroblast	XPA-/-	NER	XPA, PARP1	Inhibited	PARP1 activity	(Fang et al., 2014)
Parp1 -/- and WT MEFs, MCF7	Starvation	BER	PARP1	Inhibited	PARP1 activity	(Rodríguez-Vargas et al., 2016)
HL1 mouse cardiomyocyte	Serum and Glucose deprivation	BER	OGG1	Activated	Detection of γH2AX, p-ATM, NBS1 and 8-oxoG	(Siggens et al., 2012)
MLE-12	Hyperoxia	BER	OGG1	Inhibited	Comet tail length, OGG1 activity	(Ye et al., 2017)
U2OS	5-FU	BER	MSH2	Activated	PARP1 activity	(SenGupta et al., 2013)
C. elegans	5-FU	BER	MSH2, MSH6	Activated	RPA-1 filament formation, CHK-1 phosphorylation	(SenGupta et al., 2013)
AGS, NCI-N87	5-FU, AT101	BER	APE1	Activated	N.D.	(Wei et al., 2016)
HCT116, HEC59	6-thioguanine (6-TG)	MMR	MLH1, MSH2	Activated	PARP1 activity	(Zeng et al., 2007)
HCT116	6-thioguanine (6-TG) and 5-fluorouracil (5-FU)	MMR	MLH1	Activated	CHK-1 phosphorylation	(Zeng et al., 2013)
CAOV-3	Cisplatin	HR	BRCA2	Activated	Comet tail length	(Wan et al., 2018)
786-O	Sunitinib	HR	RAD51	Activated	Micronuclei formation	(Yan et al., 2017)
Mouse Oocytes	Rad51 RNAi	HR	RAD51	Activated	Comet tail length	(Kim et al., 2016)
CNE-1, CNE-2	Ionizing radiation (IR)	HR	RAD51	Activated	Detection of γH2AX	(Mo et al., 2014)
HT-29, DLD-1	Ionizing radiation (IR)	NHEJ	UVRAG	Activated	Detection of γH2AX, nuclear foci positivity of 53BP1	(Park et al., 2014)
TLR4mut liver	Diethylnitrosamine (DEN)	NHEJ	Ku70	Inhibited	Detection of γH2AX and 8-oxoG	(Wang et al., 2013)
Sqstm1 -/- and WT MEFs	Ionizing radiation (IR)	NHEJ	FLNA and RAD51	Activated	Detection of γH2AX and TP53BP1	(Hewitt et al., 2016)
Bone marrow, Hematopoietic cells	Ionizing radiation (IR)	HR, NHEJ	N.D.	Activated	Detection of γH2AX, Comet tail length	(Lin et al., 2015)
L2A -/-; Atg7 -/- and WT MEFs	Etoposide	HR, NHEJ	CHK1	Inhibited	Detection of γH2AX, Comet tail length	(Park et al., 2015)
Atg7-/- and WT MEFs	Ionizing radiation (IR)	HR, NHEJ	CHK1	Activated	Detection of γH2AX, Comet tail length, Plasmid-based NHEJ and HR assay	(Liu et al., 2015)

## 5. Modulation of DNA repair pathways by autophagy

Involvement of autophagic protein and/or activity in NER have been reported. NER activity has been documented to be reduced in autophagy-deficient cells. Twist1, an oncogenic transcription factor, has also been shown to modulate the NER activity through transcriptional regulation of XPC. In addition, accumulated SQSTM1/p62 stabilizes Twist1 and further leads to inhibition of p300 which is one of the crucial factors for DNA damage recognition by DDB2 upon loss of autophagy (Qiang et al., 2016). Of note, autophagy and proteasome cooperatively regulate the stability of this transcription factor (Qiang et al., 2014). In another study, the downregulation of UVB-induced DNA repair activity and XPC expression has been associated with the absence of another autophagy-associated protein AMPK (Wu et al., 2013). Therefore, the transcriptional involvement of autophagy has been documented to affect the global NER pathway. In contrast, the involvement of autophagy proteins in NER has not necessarily been linked with their role in the autophagic activity. UVRAG, is a component of VPS34 complex following UV-induced damage, promotes the assembly of DDB2-DDB1-Cul4A-Roc1 (CRL4DDB2) ubiquitin ligase complex and histone modifications upon DNA damage were found to be regulated by this ubiquitin ligase complex. In line with this, complex-assisted modification of histone leads to the recruitment of XPC proteins to the lesion site for NER activity. Although UVRAG modulates NER activity, autophagy deficiency or inhibition of autophagic flux has shown to be unable to prevent UV damage induced by UVRAG deficiency which suggests an autophagy-independent role of UVRAG.

XPA, a key protein in the NER pathway, has been linked with autophagy modulation upon DNA damage and implicated in chemo-resistance and neurodegeneration in an autophagy-dependent manner. Silencing of XPA has been shown to sensitize melanoma cells against cisplatin following autophagy impairment through activation of PARP1 (Ge et al., 2016). Loss of XPA in patient tissues represented mitochondrial dysfunction and impaired mitophagy, presumably due to PARP1 hyperactivation and reduced activity of NAD^+^-SIRT1-PGC-1α-UCP2 pathway (Fang et al., 2014; Scheibye-Knudsen et al., 2014).

Most of the studies released on autophagy and SSB repair concepts relies on the recovery of ROS-induced DNA damage. Due to the highly reactive nature of ROS, ROS are considered as the primary reason for base alterations and subsequent activation of BER. Although the activation of BER is vital for base alterations caused by ROS, the crosstalk between BER and the autophagy mechanism has not been fully understood yet. PARP1, a critical BER enzyme, resides at the nexus of autophagy and BER pathways and acts as a regulator in both cancer and cell death. Therapy-induced increase of PARP1 activity has been associated with resistance to cell death and prolonged cancer cell survival (Ménissier de Murcia et al., 2003). AMPK senses cellular ATP levels and ATP depletion leads to restraining the capacity of DNA repair through reducing the activity of PARP1 and triggering autophagy (Rodríguez-Vargas et al., 2016). Increased autophagic activity upon nutrient starvation decreases protein levels of OGG1 glycosylase and further impairs BER in cardiomyocytes. Moreover, autophagic activity did not affect other BER enzymes including, PARP1 and APE1 under this condition (Siggens et al., 2012). 

In another study, a high level of oxygen exposure results in ROS-related DNA damage, accumulation of OGG1 protein and increased inflammatory markers. Hyperoxia-induced DNA damage regulates autophagy by an OGG1-assisted transcriptional increase of Atg7. Moreover, OGG1 was documented as an autophagic target where it represents a gas and brake model for cells upon DNA damage (Komakula et al., 2018). BER-associated AP endonucleases are an important player for the activation of both repair and autophagy in model organisms following 5-FU-dependent DNA damage (SenGupta et al., 2013). Inhibition of BER-associated AP endonuclease APE1 by specific inhibitors prevents gastric cancer resistance which was found to be linked with prosurvival autophagy in the presence of 5-FU (Li et al., 2016). BER enzymes of *C. elegans* , APN-1 and EXO3 operate in the same pathway and induce 5-FU toxicity, initiate DDR and trigger autophagic cell death. Therefore, autophagic activity regulates crosstalk between BER and MMR in the presence of DNA damage.

Replication stress, mainly attracted by MMR, is mostly associated with the autophagic activity. Moreover, MMR was also shown to reduce the activity of HR which targets mostly replication stress-caused damage (Robison et al., 2004). Therefore, understanding the role of MMR and autophagic activity is quite crucial for replication stress-assisted damage. Up to now, no direct interaction has been shown between autophagy and MMR system, yet MMR system was found to be essential for autophagy induction against various chemotherapeutic agents including the nucleoside analogs 6-thioguanine (6-TG) and 5-fluorouracil (5FU) (Zeng and Kinsella, 2010). 

Studies conducted on an isogenic couple of MMR-deficient and MMR-active cancer cells revealed that only MLH1 and MSH2 active cells, which both have a role in MMR, able to induce autophagy upon 6-TG and 5-FU treatment. Moreover, p53 status was also found to be associated with this phenomenon and loss of p53 able to block subjected autophagic induction (Zeng and Kinsella, 2007).

So far, in this review, we discussed the mechanism of autophagy, DNA damage, DDR and SSB-dependent repair mechanisms. In addition, DSB is another major type of damage to DNA. The role of autophagy on HR mechanisms has been widely studied. Most of the studies conducted using autophagy-deficient cells to establish their connection. In line with this, both the proficiency of HR and autophagic activity is known to be affected by cell cycle stage and progression. Therefore, revealing new connections between those distinct mechanisms is vital for understanding genomic maintenance better.

BRCA2 protein is a key mediator of HR, which exerts its action through disassembling native Rad51 heptamers and promoting the loading of Rad51 monomers onto ssDNA replacing RPA (Mladenov et al., 2016). BRCA2 deficient cancer cells exhibit sensitive phenotype against cisplatin compared to normal counterparts (Sakai et al., 2008; Rytelewski et al., 2014). In addition, both absence of BRCA2 and autophagic activity further promote the efficacy of cisplatin (Wan et al., 2018). In line with this autophagic protein Beclin1 expression level was found to be higher in BRCA1 positive tumors compared to the negative ones (Li et al., 2010). Moreover, Beclin1 and BRCA1 are two genes that reside on close approximation of the same chromosome 17. The deletion of both or only BRCA1 deletion has been associated with the development of breast and ovarian cancers ( Laddha et al., 2014).

During cell division, some of the chromosomes cannot be incorporated into the nucleus or are damaged which induces the establishment of extranuclear bodies called micronuclei. Moreover, micronuclei may simply arise from unrepaired DSBs due to the dysfunction of DSBs specific repair mechanisms (Fenech et al., 2011). Rather than replication stress some of the genotoxic agents may also induce micronuclei formation. Studies showed that autophagic activity increased parallel under the micronuclei formation circumstances. Micronuclei have shown to be surrounded by autophagy marker LC3 protein which can be subjected to autophagic degradation (Rello-Varona et al., 2012; Sagona et al., 2014). For instance, Sunitinib, a multitargeted receptor tyrosine kinase (RTK) inhibitor, caused the formation of micronuclei and increased autophagic activity in renal cancer cells. DNA damage-associated proteins RAD51 and PARP1 are required for the clearance of these micronuclei caused by sunitinib. Deprivation of both RAD51 and PARP1 proteins alleviates sunitinib-induced autophagy and further formation of basal micronuclei (Yan et al., 2017).

RAD51, is an important homologous recombination protein in the repair of DSBs. Most of the autophagy-associated signaling molecules including ERK1/2 and Akt, are reported to alter the expression of RAD51 which adversely affects the autophagic process (Golding et al., 2009; Ko et al., 2016; Kim et al., 2016). Oocyte meiosis is found to be disrupted by silencing of Rad51 which resulted in increased DNA damage including defective chromosome segregation and spindle assembly. Moreover, loss of Rad51 is linked with damaged mitochondria and decreased ATP production. 

Concomitant activation of autophagy facilitates the clearance of Rad51-assisted accumulation of damaged mitochondria (Kim et al., 2016). A reduced level of RAD51 is associated with enhanced radiosensitivity followed by autophagic inhibition (Mo et al., 2014). In accordance with this data, autophagy-deficient cells exhibit impairment in the downstream recruitment of homologous recombination repair proteins including BRCA1, UIMC1/RAP80, RAD51 and alleviated chromatin ubiquitination triggered by irradiation. Knocking down of autophagy receptor protein SQSTM1 is found to rescue the phenotype which implied that autophagic deficiency-caused alleviation in the recruitment of DNA repair factors regulated by SQSTM1 (Feng and Klionsky, 2017).

All the above-mentioned studies stated elaborate connections between HR and autophagy. In particular, some of the well-known chemotherapeutic agents were found to support these intricate connections. Thereby, the connection between HR and autophagy may be crucial in terms of cancer therapy. However, the specificity of individual repair mechanisms somehow associated with different DNA damage types and sources. So, more detailed studies need to be utilized in this context.

In line with the other SSB and DSB repair pathways, autophagy proteins and autophagy-related contexts are also associated with NHEJ mechanisms. For instance, radiation-induced DNA damage was associated with the abundance of cellular UVRAG level. Moreover, they showed that UVRAG has a direct function on step-by-step activation of DNA-PK by facilitating the recruitment of DNA-PK to damaged DNA ends and led to the formation of the Ku-DNA-PKcs complex (Zhao et al., 2012). Silencing of Beclin 1 or UVRAG may enhance radiation-induced DSBs and initiate cell death in colorectal cancer. Moreover, knockdown of Beclin 1, UVRAG, and ATG5 increase radiation-induced 53BP1, but not RAD51 which supports NHEJ, not HR (Park et al., 2014).

Another function of NHEJ has been linked with one of the most lethal and prevalent cancers, hepatocellular carcinoma (HCC). Oxidative stress and following chronic liver damage were shown to be associated with HCC. The involvement of NHEJ has been considered in this context as well. DEN (diethylnitrosamine) exposure was shown to cause the accumulation of both ROS and damage-associated molecular patterns (DAMPs) which may further lead the genomic instability and hepatocyte transformation. Interaction of DAMPs with TLR4 founds to activate an immune response against liver injury and may trigger both autophagy and senescence. Autophagic activity was reported to block the malignant transformation of hepatocytes under these circumstances. The key NHEJ players which have a critical function in DSB are XRCC6/Ku70 and XRCC5/Ku80. In addition, mutation of TLR4 is associated with a decreased level of XRCC5-XRCC6 protein expression upon DEN. Hence, decreased XRCC5-XRCC6 expression leads to continual DNA damage and ROS related ER stress in TLR4 mutant liver. XRCC6 was found to enhance the expression or activity of DNA-dependent repair kinase complex ATM-PRKDC (DNA-PKcs) along with PARP1 and TP53, which together modulate autophagy and apoptosis in hepatocytes (Wang et al., 2013).

Irradiation (IR) led to inhibition of cell proliferation, induction of apoptosis and DNA damage in hematopoietic stem cells (HSCs). Moreover, autophagy served as a prosurvival mechanism upon IR in HSCs. Autophagy deficiency in HSCs was associated with the absence or reduction of DNA damage regulatory proteins upon IR, which are both critical in HR and NHEJ mechanisms contradictory to its role in cellular clearance. Besides, autophagy either facilitates the degradation of DNA damage inhibitory proteins or leads to the inhibition of proteasomal degradation of DNA damage proteins. For instance, previous studies have shown that mTOR inhibition increases the levels of XRCC4 and Ku80 proteins, whereas autophagic deficiency reduces the levels of these proteins. Thus, autophagy and its clearance role have been tightly associated with IR-induced DNA repair in HSCs (Lin et al., 2015). In line with this, rather than macroautophagy, another autophagic degradation system called chaperone-mediated autophagy (CMA) may be another crucial player in this context. Inhibition of autophagy led to increased CMA activity which was responsible for the degradation of important DDR protein, checkpoint kinase 1 (Chk1) (Park et al., 2015). Chk1 reported the intersection between HR and NHEJ DSB mechanisms. Studies revealed that loss of autophagic activity impaired HR and favored the NHEJ in autophagy-deficient cells. Consequently, further inhibition of DNA-damage induced NHEJ led to severe genomic abnormalities and cell death in the absence of autophagy (Liu et al., 2015).

SQSTM1/p62 is a receptor protein which degrades upon autophagic activity. Rather than cargo association, p62 serves several different roles in cells especially upon oxidative stress conditions. For instance, p62 may shuttle in between cytosol and nucleus upon oxidative stress to facilitate Nrf2-dependent antioxidant response (Komatsu et al., 2010). Not surprisingly, as a stress-responsive molecule, it is also related to DNA damage foci and offers a nuclear role for p62. Upon DNA damage, p62 was reported to interact with Filamin A, which normally modulates the recruitment of RAD51 on DSBs, and regulates their proteasomal degradation to favor NHEJ rather than HR. Of note, p62 degradation by autophagic activity restrains this phenomenon and favors HR over NHEJ as well (Hewitt et al., 2016).

## 6. Conclusion

Maintaining cellular homeostasis requires a fine-tuning between stress and stress-response mechanisms. Dysregulation of any of these mechanisms may impair vital cellular mechanisms including autophagy, DDR and DNA repair. Exposure of several stresses such as nutrient deprivation and DNA damage led to the activation of autophagy and DNA repair mechanisms to avoid lethal events e.g., genomic instability. Therefore, it is quite important to understand both these pathways and their interactions under certain conditions. As summarized in this review, DNA repair and autophagy mechanisms have been shown to cooperate in many different aspects.

Autophagy is modulated by distinct protein complexes at different stages and tightly controlled by several cellular modulators. On one hand, individual autophagy proteins are associated with DNA repair-related proteins in the presence of DNA damage. On the other hand, altered autophagic activity upon DNA-damage was found to affect the cellular DNA repair capacity. Not surprisingly, the function of autophagy in cellular protein clearance, e.g., targeting a protein that has a role in one specific repair pathways, may also involve in the decision of DNA repair mechanism in a context-dependent manner.

Modulation of these pathways are under consideration in the treatment of a spectrum of diseases, including degenerative diseases and cancer. For instance, DNA damage causing chemotherapeutics are widely accepted agents to treat cancer in the clinic. In general, autophagy is strictly involved in the mechanism of the action of these agents. Balancing autophagy under these circumstances is found to alter the efficacy of the treatment in many cases. Therefore, a comprehensive understanding of the crosstalk between autophagy and DNA repair might contribute to the efforts involving both modulations as an innovative treatment approach. Future studies are expected to identify additional factors that modulate both processes including noncoding RNAs e.g., miRNAs which we previously described in our reviews (Kocaturk et al., 2019; Akkoc and Gozuacik, 2020). Although, there is no study directly showing the intersection. In this review, we covered all presented data showing interactions between autophagy and DNA repair and discussed further potential associations.
